# In Vitro Cultivation and Ginsenosides Accumulation in *Panax ginseng*: A Review

**DOI:** 10.3390/plants12173165

**Published:** 2023-09-03

**Authors:** Fengjiao Xu, Anjali Kariyarath Valappil, Ramya Mathiyalagan, Thi Ngoc Anh Tran, Zelika Mega Ramadhania, Muhammad Awais, Deok Chun Yang

**Affiliations:** 1 Graduate School of Biotechnology, College of Life Sciences, Kyung Hee University, Yongin-si 17104, Gyeonggi-do, Republic of Korea; fengjiaoxu96@gmail.com (F.X.); tranngocanh@khu.ac.kr (T.N.A.T.); zelika.mega@unpad.ac.id (Z.M.R.); awaiskazmi@khu.ac.kr (M.A.); 2 Department of Biopharmaceutical Biotechnology, College of Life Science, Kyung Hee University, Yongin-si 17104, Gyeonggi-do, Republic of Korea; anjalikv111@khu.ac.kr (A.K.V.); ramyabinfo@gmail.com (R.M.)

**Keywords:** *P. ginseng*, in vitro tissue culture, ginsenosides accumulation, ginseng breeding

## Abstract

The use of in vitro tissue culture for herbal medicines has been recognized as a valuable source of botanical secondary metabolites. The tissue culture of ginseng species is used in the production of bioactive compounds such as phenolics, polysaccharides, and especially ginsenosides, which are utilized in the food, cosmetics, and pharmaceutical industries. This review paper focuses on the in vitro culture of *Panax ginseng* and accumulation of ginsenosides. In vitro culture has been applied to study organogenesis and biomass culture, and is involved in direct organogenesis for rooting and shooting from explants and in indirect morphogenesis for somatic embryogenesis via the callus, which is a mass of disorganized cells. Biomass production was conducted with different types of tissue cultures, such as adventitious roots, cell suspension, and hairy roots, and subsequently on a large scale in a bioreactor. This review provides the cumulative knowledge of biotechnological methods to increase the ginsenoside resources of *P. ginseng.* In addition, ginsenosides are summarized at enhanced levels of activity and content with elicitor treatment, together with perspectives of new breeding tools which can be developed in *P. ginseng* in the future.

## 1. Introduction

*Panax* species, commonly referred as ginseng, which belong to the Araliaceae family, are slow-growing perennial herbal medicines with adaptive properties [1]. The word ‘Panax’ comes from the Greek word ‘pan’ (all) and ‘zxos’ (treatment of medicine), which means cure-all [2]. There are 15 species in the Panax genus, and they are listed in Table 1 [3,4]. Among these, there are three commonly used commercial ginseng species, including *Panax. ginseng*, *P. quinquefolius*, and *P. notoginseng* [5]. Most of the secondary compounds, especially ginsenosides, have been recorded in the roots. They act as tonic agents and stimulants that have been used in Asian countries for thousands of years, and they are becoming increasingly popular all over the world [6]. Pharmacological studies have demonstrated that ginseng species are rich in bioactive compounds such as ginsenosides, polysaccharides, flavonoids, phenolics, and volatile oils [7]. Among them, ginsenosides are known as the main bioactive ingredients responsible for the pharmaceutical efficacy of ginseng species [8], such as their anti-cancer [9], anti-fatigue [10], anti-inflammatory [11] activity and their prevention of cardiovascular disease [12], obesity [13], and cerebrovascular diseases [14], etc.

The increasing demand for herbal remedies has escalated the market value of ginseng species, but it has also created huge challenges for industries and governments to standardize and regulate plant-derived natural products to ensure consumer safety [15]. To address these issues, good standardized guidelines of agricultural cultivation should be established. For example, the Government of Canada established the Natural Health Products Directorate (NHPD) to enact the new legislation (JUS-601727) to govern the manufacture and marketing of natural health products [16].

However, the prolonged cultivation period, susceptibility to pathogens and replant diseases, limited availability of arable land, and labor-intensive cultivation practices have impeded farmers from meeting the growing market demand [15]. Moreover, the use of pesticides and the fluctuating environmental conditions resulting from global warming have compelled researchers and plant scientists to explore alternative methods to meet the demands of a rapidly increasing population [17]. Traditionally, there are two sources for obtaining ginseng species, one of which involves harvesting wild ginseng species. However, due to the over-exploitation of wild ginseng species and the destruction of arable land for growing ginseng species, the amount of wild ginseng is decreasing [18]. Another origin of ginseng species supply is to grow it in fields or forests, which is a time-consuming and labor-intensive process [19]. Furthermore, replanting disease will also result from intensive replanting, where replanting a second time in the same place will often lead to failure [20]. For these reasons, ginseng is becoming increasingly difficult to obtain and more expensive.

**Figure 1:**
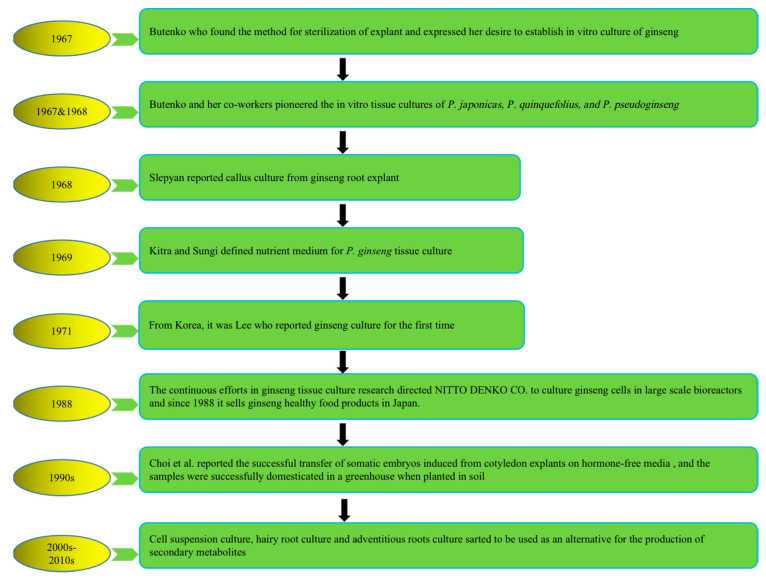
History of in vitro plant tissue culture of ginseng species. Note: This figure shows the in vitro cultivation of ginseng species from 1967 to present.

To address the above problems, tissue culture approaches have developed rapidly in recent years to produce bioactive compounds with high content and activities that not only have health-promoting properties but also significantly alter natural sources. The first attempt at plant cell cultivation was by the Austrian botanist Haberlandt in 1902, who isolated plant cells and cultivated them outside the whole plant [21]. The successful development of a nutrient medium by Murashige and Skoog in 1962, commonly known as MS medium, has remained in use, with minor adjustments [22]. The introduction of this specific nutrient medium, along with a range of plant growth regulators (PGRs), has significantly revolutionized the field of plant tissue culture research, leading to its successful integration as a viable commercial venture offering numerous advantages and possibilities. Multiple investigations have subsequently demonstrated that undifferentiated plant cells, such as calluses and cell suspensions, can be a valuable resource for producing identical secondary metabolites found in naturally occurring plants. It represents a significant advancement in plant research, over a century after Haberlandt’s initial attempts in the field [23]. Plant tissue culture technology is helpful for plant transformation, clonal propagation, breeding, and protection of pharmaceutical plants and crops. Figure 1 describes the history and establishment of ginseng species’ in vitro plant tissue culture [24,25,26,27,28,29,30].

**Figure 2:**
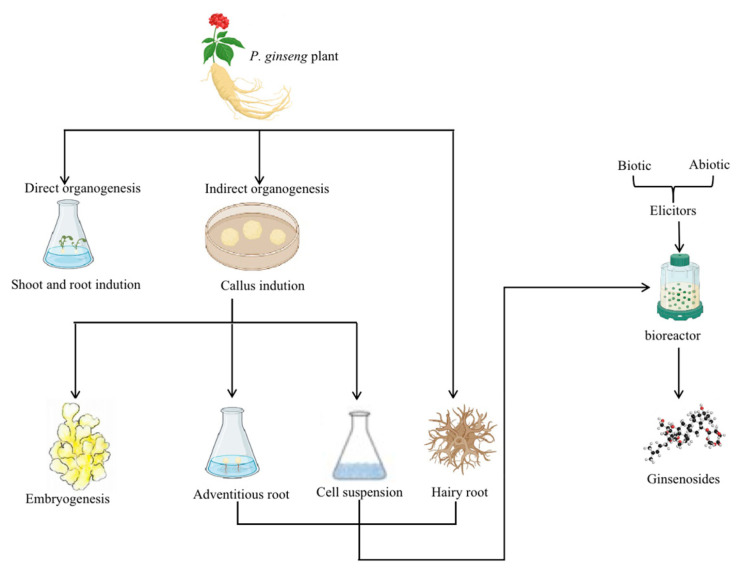
A summary of the standard procedures of *P. ginseng*’s in vitro culture methods and ways to increase ginsenosides accumulation. Note: The direct and indirect organogenesis methods can obtain the in vitro culture of *P. ginseng*. Likewise, the accumulation of ginsenosides can be acquired through the adventitious roots, cell suspension, and hairy root cultures. In addition, the large-scale culture in bioreactors treated with biotic and abiotic elicitors also increases the biomass and the ginsenosides accumulation.

Recent research shows that in vitro tissue culture methods produce ginsenosides successfully from *P. ginseng* [31]. Therefore, this review summarizes the in vitro tissue culture methods on *P. ginseng*, which include direct root and shoot induction organogenesis without the formation of callus and indirect organogenesis from callus for further embryogenesis. In addition, in vitro biotechnological methods for ginsenosides accumulation include adventitious roots culture, cell suspension culture, hairy root culture, and bioreactor culture for large-scale propagation, which are also discussed, as depicted in Figure 2. Furthermore, this review discusses specific plant breeding methods that open new opportunities for ginseng breeding activities.

## 2. In Vitro Culture of *P. ginseng* Technologies

**Figure 3:**
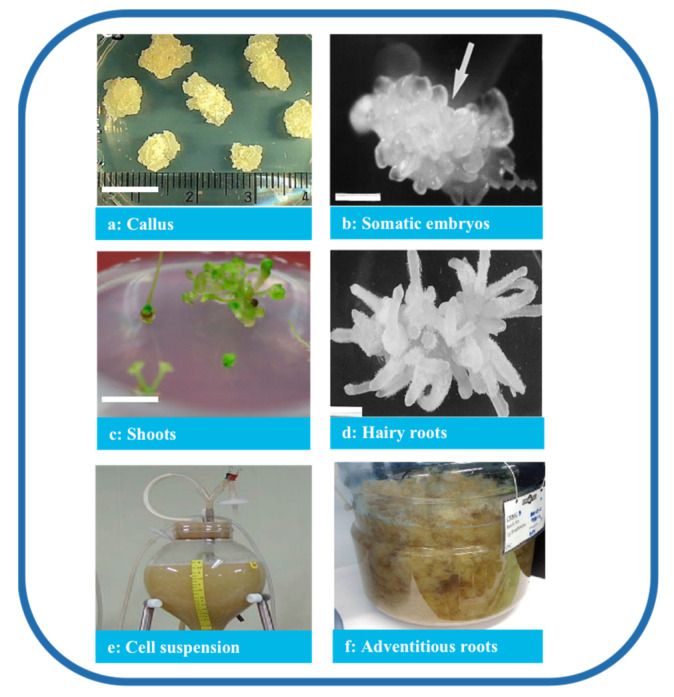
Types of *P. ginseng* tissue culture. Note: (**a**) Callus (bar 1 cm) [32], (**b**) somatic embryos (bar 1 mm) [33], (**c**) shoots (bar 1 cm) [32], (**d**) hairy roots (bar 820 μm) [34], (**e**) cell suspension [35], and (**f**) adventitious roots [36].

Tissue culture is classified based on the purpose of the culture and the source of materials. Several processes were established in *P. ginseng* based on the organization of the cells and organs (Figure 3), producing a consistent quality of *P. ginseng* and promoting the sustainable application of the species. In addition, under controlled culture conditions, numerous factors influence the quantity and quality of ginsenosides, such as medium constituents, pH, light conditions, culture temperature, explants, and abiotic factors.

### 2.1. Direct Organogenesis of P. ginseng

Direct organogenesis is the induction of roots and shoots directly from explants without forming a callus. Shoot culture demonstrated genetic stability and the potential to produce secondary metabolites. However, the research on the direct organogenesis of ginsenoside production is limited. Among the limited research available, it is vital to discuss the work of Hee-Young Lee et al., who studied the regeneration of *P. ginseng* from embryos obtained from the cultures of anthers. The results from the study indicated the optimum conditions required for the regeneration of *P. ginseng*—for example, cold treatment matters. The highest callus induction rate was achieved when the explants were cultured post-pretreatment at 4 °C.

On the other hand, the findings also report that the application of PGRs also affects shoot and root production. The shoots and roots can be induced on a medium supplemented with Gibberellin A3 (GA3) and 3-Indolebutyric acid (IBA) at the concentration of 28.9 μM and 14.7 μM, respectively [32]. Another study suggested that supplementing naphthaleneacetic acid (NAA) and IBA enhances the organogenic potential. Though IBA attained the highest shoot and root production rates, the roots induced by NAA showed better growth and were thicker than those of IBA. In addition, the roots induced by NAA also attained the highest ginsenosides production rates [37].

### 2.2. Indirect Organogenesis of P. ginseng

Indirect organogenesis, called callogenesis, is regenerating plantlets from the callus. The morphology and characteristics of calluses also influence organogenesis and biomass production. Friable and compact calluses are the two types of callus used in suspension culture and regeneration research, respectively [38].

#### 2.2.1. Callus Culture

To date, explants, such as roots, stems, seeds, leaves, buds, petioles, anthers, and hypocotyls, have been used to induce ginseng callus. Among them, the leaves and roots are the most common ones. Typical callus induction and culture are carried out using Murashige and Skoog’s (MS) basic medium or Gamborg medium (B5) with 3% sucrose and various PGRs at different concentrations. Researchers have investigated the effects of PGRs, among which 2,4-Dichlorophenoxyacetic acid (2,4-D) is the most potent one for the induction of the callus of many plant species. A summary of callus cultures is given in Table 2. Generally, the ginseng explants are cultured in the dark at 23 ± 2 °C. Wang et al. successfully induced callus from *P. ginseng* roots using MS medium supplemented with 2 mg/L of 2,4-D and 0.5 mg/L of Kinetin (KT) [39]. Similarly, Chang et al. [40] induced callus from ginseng roots using MS medium enriched with 1 mg/L of 2,4-D. However, the growth of the callus was initially slow, with only 1 cm of elongation in diameter after ten weeks. Nevertheless, it grew vigorously when the callus was subcultured on a new medium at 6–8-week intervals. In another study, Liu et al. used 3-year-old fresh ginseng roots as explants to induce callus on a modified MS medium enriched with 2 ppm of 2,4-D, 0.5 ppm of thidiazuron (TDZ), and 1 g/L of peptone [41]. They also induced another callus from 2-year-old ginseng roots on MS medium supplemented with 1 mg/L of 2,4-D and 0.1 mg/L of KT, sub-culturing every 15 days. As a result, after six months, they obtained three types of calluses [42].

#### 2.2.2. Somatic Embryogenesis of *P. ginseng*

Using somatic embryogenesis for propagation allows a quick propagation of the superior ginseng lines while decreasing the variability commonly associated with seed propagation. The first and most crucial step in this process is the transition of somatic cells to embryonic cells. Somatic embryogenesis involves de-differentiating somatic cells into totipotent embryonic stem cells, which can produce embryos under appropriate in vitro conditions, ultimately developing into a whole plant [43,44]. Several studies have been conducted in ginseng to explore and optimize the conditions necessary for successful somatic embryogenesis [45].

In the next stage, they will develop into a whole plant after somatic embryogenesis. In *P. ginseng*, the first observation of somatic embryogenesis was reported in the callus derived from the roots by Butenko [25]. Since then, the regeneration of plants has been achieved through somatic embryogenesis using ginseng calluses derived from roots [40,46], zygotic embryos [47], somatic embryos [48], and protoplasts [49] isolated from somatic embryos (Table 3). The basic medium provides the nutritional composition and necessary elements for the growth and development of the explants. Most studies have employed MS medium for callus formation, proliferation, and somatic embryogenesis. In addition, Schenk and Hildebrandt medium (SH) and B5 have seldom been used for shoot regeneration and embryoid formation [50,51]. The in vitro propagation of somatic embryos in *P. ginseng* has been achieved using 2,4-D, KT, and NAA, but their concentrations and combinations vary depending on the type of explants.

Somatic embryo germination requires either chilling treatment for 8 weeks or GA3 hormone treatment at concentrations over 1.0 mg/L. Ultrastructural observation of cotyledon cells showed that without the treatment of chilling or GA3, somatic embryos contained large amounts of lipid reserves, dense cytoplasm, proplastids, and inactive mitochondria. Conversely, after chilling or GA3 treatment, the well-developed chloroplasts and functioning mitochondria with multiple cristae were seen in somatic embryos, indicating they may enter dormancy after maturation, similar to zygotic embryos. Recent studies have reported over 80% plant survival in hybrid ginseng, achieved by culturing embryos on GA3-supplemented medium, transferring them to hormone-free 1/2 SH medium, treating developed taproots with GA3 to break shoot dormancy, and transferring them to the soil. Therefore, GA3 pretreatment is crucial for successful transplantation [52].

Other factors, such as the salt content of the medium, also play a crucial role in somatic embryo induction. Choi et al. studied the effects of macrosalt stress on the embryogenesis of *P. ginseng*. The results showed that the highest frequency of somatic embryogenesis was observed on a medium containing 61.8 mM NH_4_NO_3_ with a ratio of NH_4_^+^:NO_3_^−^ at 21:39. Among the test media, including MS, B5, and SH, the maximum formation rate of the somatic embryo was observed when cotyledon explants were cultured on 1% agar MS medium with the supplementation of sucrose at 5% [29,53].

Different attempts have been made to regenerate ginseng through tissue culture using somatic embryogenesis techniques [54,55]. However, most regenerated plants cannot survive when transferred to soil. Shoot or multiple shoot formation has been successful from somatic embryos. However, taproots cannot be obtained, as the reproductive capacity of the multi-shoot complex gradually decreases and eventually disappears during long-term subculture (over 12–18 months). This phenomenon was observed in ginseng, where a single somatic embryo can regenerate into a plant with well-developed roots and shoots.

**Figure 4:**
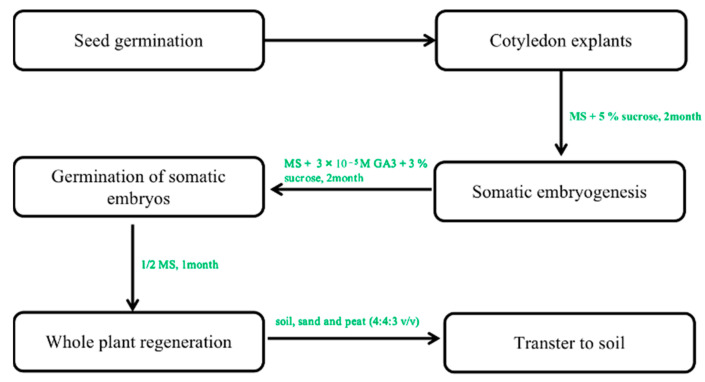
The protocol of somatic embryogenesis from callus to whole plants. Note: This figure shows the indirect organogenesis. First, the optimal callus was selected to form the embryogenic callus. In the next step of somatic embryogenesis, a regenerated whole plant can be obtained under optimal culture conditions.

In contrast, multiple fused somatic embryos result in multiple shoots [50]. Only the study by Choi et al. [29,30] reported the successful transfer of somatic embryos induced from cotyledon explants on hormone-free media at 12–66% frequency in the regenerated plant. In his study, ginseng plants with well-developed shoots and roots regenerated from single embryos were successfully domesticated in a greenhouse when planted in soil (Figure 4) [56]. This regeneration protocol is very effective in the induction of whole plants.

Furthermore, optimal physical conditions, including light, temperature, and relative humidity, were identified for the in vitro propagation of ginseng species. A photoperiod of 14–16 h per day, with a cold white fluorescent lamp providing a light intensity of 24–80 μmol m^−2^ s^−1^, and a temperature of 23 ± 2 °C were found to be appropriate for the incubation and maintenance of cultures [33,53].

In addition, there is a desperate need for a reliable and fast method to propagate the superior chemotype of ginseng species. For this purpose, it is crucial to establish a fully controlled in vitro micro-saline environment using shakers, temporary soaking, or bioreactors, which will enable the production of healthy and uniform seedlings. Additionally, molecular-marker-assisted protocols are highly recommended for verifying clonal fidelity and ensuring the production of identical clones. A well-known barrier to the effectiveness of plant production is the deterioration of culture vigor and regenerability over time. Various phenotypes, such as changes in plant height, biomass, grain yield, resistance to disease and pests, acid and salt tolerance, and agronomic performance, have all been linked to somaclonal variation. Over the 20 years of ginseng cell subculture, ginsenosides comprised just around 0.024% of the dry weight [57]. Raul Sanchez-Muñoz et al. indicated that the fundamental obstacle in creating commercially viable plant biofactories appears to be the alterations in methylation patterns, the primary mechanism predicted to be implicated in yield loss over time [58]. Therefore, clone maintenance should be investigated further for stable biomass and secondary metabolites production.

**Table 3:** List of the common conditions for somatic embryogenesis of ginseng.

Explants	Medium	PGRs	Embryogenesis Rate	Other Factors	Ref.
seeds	MS	2,4-D+ kinetin/ hormone free	45%/32.5%	Most of the single embryos were formed on a hormone-free medium, but multiple embryos were formed on a hormone-containing medium.	[50]
cotyledons	MS	2,4D+BA+ lactalbumin hydrolysate	87%	The use of glucose can enhance somatic embryo formation.	[59]
cotyledon	MS	61.8 mM of NH_4_NO_3_	56.3%	The highest frequency of somatic embryo formation occurred in the following order: NH_4_NO_3_ > KNO_3_ > KH_2_PO_4_ > MgSO_4_ > CaCl_2_.	[29]
zygotic embryos	MS	2,4-D+ kinetin	NM	NM	[60]

NM: not mentioned.

## 3. Ginsenoside Biosynthesis and Biotechnological Production

### 3.1. Biosynthetic Pathways of Ginsenosides

Ginsenosides are triterpenoids or saponins, secondary metabolites with significant medical value, particularly in the pharmaceutical industry. Because they resemble steroidal hormones, these secondary metabolites have a variety of pharmacological characteristics. According to the aglycone structure, ginsenosides are classified into dammarane or oleanane types. Ocotillol-type ginsenosides are derived from oleanolic acid precursors. In contrast, the dammarane-type ginsenosides can further be classified into protopanaxadiol (PPD) and protopanaxatriol (PPT) ginsenosides [61], which are the major ginsenosides in *P. ginseng* and the main bioactive constituents for its biological activities. 

**Figure 5:**
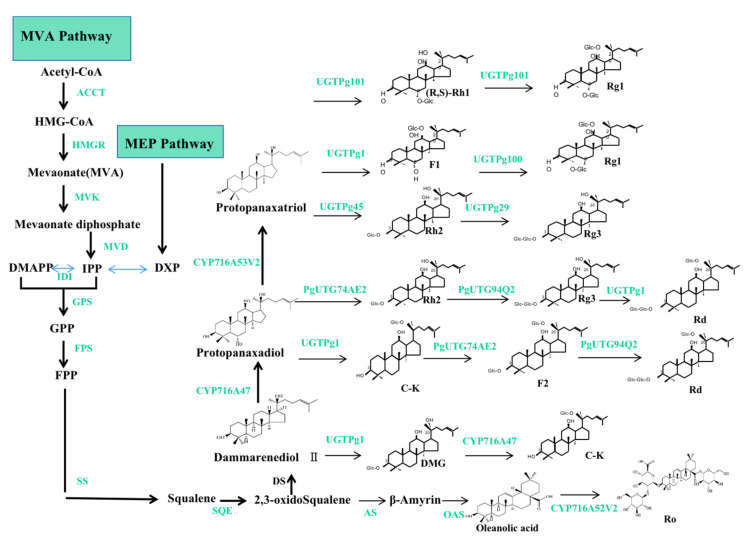
The biosynthetic pathways of ginsenosides. Note: the two pathways are MVA and MEP, respectively; the three stages are the formation of IPP and DMAPP; IPP and DMAPP are converted into 2,3-oxidoSqualene; ginsenosides of dammarenediol type; PPD and PPT types are synthesized from 3 steps. The green color represents the related genes and enzymes.

The biosynthetic pathways of ginsenosides have been demonstrated, as shown in Figure 5. Generally, two pathways and three stages are involved in the biosynthesis of ginsenosides. The production of ginsenosides occurs in the cytosol and plastids through the mevalonic acid (MVA) pathway and the methylerythritol (MEP) pathway. The three stages are as follows: (1) firstly, isopentenyl diphosphate (IPP) and its isomer dimethylallyl diphosphate (DMAPP) are produced via the MVA and MEP pathways; (2) IPP and DMAPP are then transformed into 2,3-oxidosqualene; and (3) ginsenosides (such as Rh1, Rh2, Rg1, Rg3, Rd, C-K, F2, and Ro) are created via three reaction steps from 2,3-oxidosqualene, which include cyclization, hydroxylation, and glycosylation. The biosynthetic pathways of ginsenosides consist of more than 20 steps of consecutive enzymatic reactions, including enzymes, for example: 3-hydroxy-3-methylglutaryl coenzyme A reductase (HMGR), farnesyl pyrophosphate synthase (FPS), squalene synthase (SS), squalene epoxidase (SQE), dammarenediol-II synthase (DS), β-amyrin synthase (AS), cytochrome P450 (CYP450), and UDP-glycosyltransferase (UGT) [62,63,64].

### 3.2. Ginsenosides Accumulation in In vitro Cultivation of P. ginseng

Based on the purpose and types of tissues applied for in vitro culture, *P. ginseng* culture, which has proven successful in producing secondary metabolites, was reported in adventitious roots, cell suspension culture, and hairy roots.

#### 3.2.1. Ginsenosides Accumulation via Adventitious Roots Culture

Adventitious roots culture is considered an alternative and prospective method for cell culture because of its higher biomass, production, stability in different physical and chemical environments, and higher ginsenosides production in large-scale bioreactors. The commercial-scale production of ginseng roots has been realized only in recent years in the Republic of Korea despite the first patent on ginseng root tissue culture being invented by Metz and Lang in 1966 [35]. The adventitious roots form from unusual parts such as calluses, stems, roots, and leaves. There are four discrete stages: the induction of callus, somatic embryogenesis, the formation of adventitious roots, and root elongation [65]. Optimal conditions, such as the types of explants, types and concentrations PGRs, and medium constituents, are needed in these four stages.

NAA and IBA are the two most used exogenous hormones for adventitious root induction from the callus. IBA was found to be more effective than NAA in the induction and elongation of lateral roots. Roots that were greatly elongated and slender formed on the IBA-containing medium compared to the NAA-containing medium when cultured under dark conditions [66,67]. A study reported the effect of NH_4_NO_3_ in the medium on the adventitious root induction, and the results showed that NH_4_NO_3_ free medium was better for the adventitious root formation. At the same time, it was shown to be necessary for the further elongation of post-induced adventitious roots [68].

Plant cells’ defense mechanisms can be activated to respond to the attack of pathogens and biotic and abiotic stresses, which includes the biosynthesis of secondary metabolites [69]. In order to increase the contents of the secondary metabolite “ginsenosides,” some compounds have been used as elicitors to increase the expression and critical enzymes relevant to its biosynthesis [70]. Methyl jasmonate (MJ) is the vital signaling compound involved in the biosynthetic pathways responsible for accumulating secondary metabolites [71]. Though MJ inhibits the fresh weight, dry weight, and growth ratio of ginseng roots in the in vitro culture, the production contents of ginsenosides were up to 5.5–9.7 times as high as in the untreated roots [72]. Another kind of elicitor, organic germanium, a food supplement, was used to work as an elicitor to increase biomass accumulation. When treated on the cultured roots with organic germanium at 60 mg/L, the accumulation of ginsenosides Rb and Rg and the dry biomass of adventitious roots was enhanced [73].

Owing to the high price of MJ, which limits the mass production of ginsenosides in large-scale bioreactors, the scientific community has started exploring other approaches to increase ginsenosides production in in vitro root cultures. *Endophytes* are bacterial or fungal microbes that can colonize healthy plant tissues without showing any apparent symptoms and protect their host by producing a variety of substances. Some reports have illustrated that endophytes can stimulate secondary metabolite accumulation when serving as elicitors. For example, a remarkable enhancement effect on ginsenosides accumulation was found when treating a 28-day-old adventitious root in a suspension culture of *P. ginseng* with dried mycelium of *Aspergillus niger*. Similarly, the application of this elicitor decreased the growth of the adventitious root, and the dry weight was reduced with the increasing concentrations of the elicitor [74]. An Endophyte bacterium, strain LB 5-3, from ginseng roots cultivated in the ginseng field showed the capacity to increase biomass and ginsenosides content by four times in ginseng adventitious root cultures [75]. A fungal suspension homogenate of pathogenic fungi (*Alternaria panax* Whetz) isolated from ginseng grown on the field was processed to be utilized as an elicitor. When the 30-day-old ginseng adventitious roots were treated with this fungal elicitor at the concentration of 200 mg/L for 8 days, the maximum ginsenosides accumulation content (29.6 mg/g dry weight) was obtained, and the biomass of the adventitious roots was not significantly inhibited [76].

Some other biotechnological methods, such as the induction of polyploidy and mutagenesis, can be used as alternative technologies for the enhancement of ginsenosides accumulation. A study reported that the mutagenesis induced via γ-irradiation enhanced the ginsenosides production content up to 16-fold compared with regularly cultured ginseng roots, and this study also indicated that the ginsenosides accumulation in the mutated adventitious roots is almost 1.6-fold that in of the normal roots cultivated in the ginseng field. However, it should be noted that the results of irradiation-based mutant breeding can vary. The growth of plants is impacted differently by each spectrum of γ-irradiation [77]. In another study, different concentrations of colchicine were used to treat the adventitious roots for different lengths of time to induce octoploid roots. The results showed that the total ginsenosides and Rb-group ginsenosides contents in octoploid roots were lower than that in untreated roots. However, the treated roots with colchicine contained more Rg-group ginsenosides, especially Rg1. These results indicated that polyploid adventitious roots can enhance secondary metabolite production in ginseng. Compared to the naturally tetraploid root, the fresh and dry biomasses of the octoploid adventitious roots were significantly higher [78].

Further studies are also highly desired to promote ginsenoside yields of at least the same level as those in 6-year-old ginseng roots cultivated in the field.

#### 3.2.2. Ginsenosides Accumulation via Cell Suspension

Adventitious root cultures are an alternative method for producing stable secondary metabolites; however, root cultures of some advanced plants exhibit difficulties in harvesting bioactive ingredients and slower growth [79]. Besides adventitious roots culture, cell suspension culture has been used to produce secondary metabolites in many plant species for decades, especially in pharmaceutical botany [80]. Plant cells are regarded as green factories for synthesizing medicinal components in bulk. These cells in intact tissues, such as leaf, stem, root, and callus, are difficult to cultivate in ideal production methods and limit labor-intensive culture practices on a commercial scale. However, cell culture systems offer staggering opportunities to establish batches and continuous cultures in bioreactors of commercial scale. There have been many reports on producing pharmaceutical compounds in bulk via cell suspension culture [17]. The most commonly used method, “micro-propagation,” is related to the proliferation of shoots through a semi-solid medium. While this semi-solid system has gained moderate or high success in increasing productivity and reducing the time required to propagate commercially essential materials, it is becoming increasingly important. Micropropagation via conventional techniques is usually a time-consuming method of clonal propagation. To overcome this, shaken culture methods using a liquid medium have been promoted. A liquid medium permits close contact with plant tissues to stimulate and promote the absorption of nutrients and phytohormones, thereby promoting the growth of branches and roots [81].

Research on ginseng suspension culture has mainly focused on various factors that influence cell growth and the synthesis of secondary metabolites. These factors include selecting optimal cell lines, using elicitors, and the impact of environmental and chemical factors such as light, pH, temperature, plant growth regulators (Table 4), nitrogen, carbon, and inorganic ions. Recent findings indicate that the rate of ginsenoside synthesis does not necessarily correlate with the growth rate of ginseng callus cells. As a result, a two-stage culture approach has become increasingly popular for producing secondary metabolites in ginseng cell suspension culture. This approach involves a cell growth stage followed by a ginsenoside production stage.

As mentioned above, many researchers have indicated that many physical and chemical factors can affect the production of secondary metabolites [82]. For example, optimizing the concentrations and combinations of various hormones and nutrients is frequently effective. In one study [83], the authors established a cell suspension culture system of mountain ginseng (*P. ginseng* C.A. Meyer) in an attempt to increase the production yield of ginsenosides via manipulating their culture methods and related factors, such as media strength, the concentrations and combinations of PGRs, the presence of sucrose, and the ratio of NO_3_^+^/NH_4_^−^. The maximum biomass content was obtained in the medium containing 2,4-D. However, the ginsenosides yield was much higher in the medium containing either NAA or IBA. IBA at the concentration of 7 mg/L was optimal for accelerating cell growth and saponin productivity. The level of ginsenosides, especially of the Rb group, was enhanced by adding cytokinins (benzylaminopurine (BA) at 0.5 mg/L and KT at 0.5 mg/L) despite having no effect on cell growth. The treatment of an initial nitrogen at 30 mM showed maximum cell growth and ginsenosides production. The amount of saponins will increase when the test medium has a high NO_3_^+^/NH_4_^−^ ratio. The production of saponins was highest when nitrate was the only nitrogen source; however, when ammonium was used as a sole source, it was not beneficial for saponin biosynthesis [84]. The effect of another inorganic ion, phosphate, on cell growth and saponin accumulation was tested in the suspension culture of *P. ginseng* [85]. The results showed that the vital phosphate concentration for cell growth and the optimal concentration for simultaneous production of ginsenosides were 1.04 mM and 0.42 mM, respectively.

Elicitation has proven to be a successful method for increasing the production yield of various secondary metabolites. In a study conducted by Lu et al. [86], the effects of elicitor concentration and the time of elicitor addition on ginsenoside synthesis and cell growth in *P. ginseng* cell suspension cultures were investigated. The yeast extract and the MJ were tested, and both elicitors significantly increased saponin production. The highest level of supplemental ginsenosides measured by dry weight was 2.07%, 28 times greater than the control. The optimal time to add any elicitor was found to be on the day of inoculation. The results also showed that when MJ was used as an elicitor, removing 2,4-D from the medium was recommended, as MJ interacts antagonistically with 2,4-D. In another study [87], the impacts of MJ on cell growth and saponin accumulation in 5 L bioreactor cell suspension cultures were also studied. This study reveals that when the amount of MJ was between 50 and 400 μM, the ginsenosides accumulation was enhanced; however, with the increased concentration of MJ, the growth ratios and the fresh and dry weights of cells were strongly inhibited. The highest ginsenosides yield was obtained when MJ was used at 200 μM.

**Table 4:** Cell suspension culture of *P. ginseng*.

Medium	PGRs					Cell Growth Rate	Total Ginsenosides Content	Other Factors	Ref.
	2,4-D	6-BA	NAA	IBA	KT				
MS	1 mg/L	0.5 mg/L	1,3,5,7, 9 mg/L	1,3,5,7, 9 mg/L	0.5 mg/L	10 g/L	7.29 mg/g	Nitrite of 30 nM can increase both cell growth and total saponins	[83]
MS	0.4 m/L			2.5 mg/L	0.1 mg/L	11 g/L	21.4	Inorganic phosphate can promote cell growth and enhance saponin accumulation	[85]
MS	1 mg/L						28-fold higher than control	The MS medium was supplemented with inorganic salts: nicotinic acid, pyridoxine-HCl, etc.	[86]
MS			2 mg/L	7 mg/L	0.1 mg/L	8.82 mg/g	2.9 times higher than control	The highest ginsenosides yield were obtained when 200 μM MJ was added on day 15 during incubation	[87]

However, one of the omnipresent domain obstacles is the metabolic diversity in plant cell cultures, leading to the physically and genetically unstable production of secondary metabolites. From the perspective of biological process operation, any commercial attempt would be inhibited if there is no solution to this instability before it is scaled up [88].

#### 3.2.3. Ginsenosides Accumulation via Hairy Roots

*Agrobacterium rhizogenes*, a bacterium found in soil, can induce hairy roots through genetic transformation. This process involves the genetic modification of plant cells via the plasmid T-DNA of *A. rhizogenes*, resulting in the formation of hairy roots during auxin metabolism. Research has demonstrated that hairy root cultures of ginseng have great potential for producing large amounts of biomass and ginsenosides. According to the literature, the mother plant’s ability to manufacture secondary metabolites is on par with or surpasses the hairy root cells [89,90,91]. Hairy root culture has advantages over cell suspension culture, such as inherent genetic stability. Hairy roots have a genetic stability that is one of their hallmarks.

Moreover, using the hairy root system holds immense potential for incorporating other genes besides the Ri T-DNA genes, which can alter the metabolic pathways and generate valuable metabolites or compounds [92]. In addition, the growth rate of hairy roots is usually similar to or faster than that of cell culture, and they do not necessitate the use of PGRs in the culture medium [93]. The technique of hairy root cultivation can be traced back to the 1980s and is still undergoing refinement and standardization. In the case of ginseng, Inomata et al. [94] and Yoshikawa et al. [95] reported that the hairy roots of ginseng showed a higher content of ginsenosides and swift growth, as compared to its suspension cells and adventitious root cultures.

The light conditions are crucial in hairy root culture for the higher production of ginsenosides. For example, the effect of light on the growth and ginsenosides accumulation of *P. ginseng* hairy roots induced by *A. rhizogene* A4 was studied, and the results showed that the growth and ginsenosides accumulation was higher when cultured in the dark for 1 week and then transferred into the light condition (3500 lux) for 4 weeks. The yields of ginsenosides Rg1 and Rf increased by 3.3- and 2.4-fold, respectively [96]. The effects of electronic inhibitors on ginseng’s root growth and ginsenoside content were tested by Yang et al. Ginsenoside production was higher when hairy roots were cultured in MS medium for 4 weeks and then transferred to 1/2MS medium containing ascorbic acid or 2,5-dimethylfuran for 1 week under light conditions. When investigating the effects of culture conditions on the growth and accumulation of ginsenosides, the research demonstrated that the accumulation of ginsenosides in ginseng hair roots cultured in a 20 L bioreactor was 34% higher than that in dark culture. During culture, ginseng hair roots were irradiated with ultraviolet light. Therefore, the growth of ginseng hairy roots was decreased following UV irradiation for a long time, but the accumulation of ginsenosides increased with the extension of UV irradiation time [97].

As discussed in the adventitious root culture, the method of induction of root mutagenesis can contribute to biomass and ginsenosides accumulation and hairy root culture. Studies were conducted to assess the role of *P. ginseng* hairy roots caused by ^60^Co γ-ray irradiation. After removing the apical meristem of hairy roots irradiated below 2 Krad, lateral roots were used as cell lines. Furthermore, 206 hairy root cell lines were selected with various growth rates and forms and cultured in 1/2 MS medium without hormones. Then, 10 out of the 206 samples which showed excellent growth were chosen. Among them, y-GHR 70 and y-GHR 94 showed higher growth. 

Different elicitors could be used for the high production of growth and ginsenosides. Hairy roots of *P. ginseng* established after induction with *A. rhizogenes* KCTC 2703 were cultured in liquid MS medium free of plant hormones supplemented with different concentrations of MJ and other inducers to promote ginsenoside accumulation. The results indicated that MJ significantly increased the total ginsenoside production, especially in the Rb group [98]. Seung-Yong Oh et al. studied the effects of chitin and chitosan on the production and growth of ginsenosides, and the results showed that when ginseng hair roots were cultured on 40 mg/L chitin and applied in the third week of culture, ginsenoside content and yield were the highest. The growth of ginseng hair root culture with 1 mg/L chitosan was the best, but the ginsenoside content with 30 mg/L chitosan was the highest [99]. Additional techniques, such as including Tween 80 in hairy root cultures, have significantly increased total ginsenoside production up to three-fold [100].

Various types and concentrations of salt affect the biomass and ginsenosides accumulation. A study investigated these effects on ginseng hair root growth and ginsenoside accumulation by adding different concentrations of potassium phosphate to 1/2 MS medium. The results showed that 1.25 mM potassium phosphate supplementation increased biomass and ginsenosides accumulation [101]. Another study determined the growth rate and yield of ginsenosides against NaCl in the hairy roots of *P. ginseng*. In MS liquid culture, the highest ginsenoside content and yield appeared 4 weeks after the onset of 0.1 M NaCl treatment [102]. To study the effects of inducers on the growth and biosynthesis of ginseng hairy roots, the hairy roots were treated with different concentrations of *Haliotidis concha* according to different time processes. *Haliotidis concha* supplementation increased the biomass and ginsenoside accumulation at the concentration of 10 mg/L [103].

#### 3.2.4. Large-Scale Production of *P. ginseng* via Bioreactors

Elements like sluggish growth rates, constrained planting areas, climatic dependence, and labor scarcity primarily constrain the large-scale generation of biochemical compounds with economic value using field-grown plants. Advancements in plant cell and tissue culture techniques have facilitated the creation of significant phytochemicals. These plant cell and tissue culture procedures should reduce these adverse effects.

In plant biotechnology, significant progress has been made in utilizing bioreactor cultivation as a viable and appealing approach for biomass and bioactive compound production [104]. Compared to traditional tissue culture methods, the bioreactor system offers enhanced sophistication, allowing for individualized optimization of culture conditions. Factors such as temperature, pH, oxygen, carbon dioxide concentrations, and nutrient levels in the medium can be controlled precisely. In addition, the continuous circulation of the medium further improves nutrient availability. It is also possible to speed up cell regeneration and proliferation. Thus, production time and cost could be greatly decreased, product quality could be regulated and standardized, products could be free of contamination by pesticides, and production could be carried out throughout the year without being limited by geography [105]. The engineering of ginseng adventitious roots, cell suspension, and hairy root cultures has become a leading food biotechnology in Korea, China, and Japan. There are numerous varieties of bioreactors on the market. The stirred-tank bioreactor is the type that is used most frequently. It enables simple cell collection at various phases due to its larger size and capacity to boost the amount of nutrients. Even airlift and balloon-type bubble bioreactors produce ginsenosides in large quantities because they are more effective at transporting oxygen and have accurate flow predictions, reducing the shearing of cells [62].

Different bioreactors possess various advantages, such as ginsenoside accumulation in *P. ginseng* adventitious roots, cell suspension, and hairy root culture. In one study, the effects of organic nutrients on growth, the development of biomass, and ginsenosides production from the adventitious roots of *P. ginseng* in a balloon-type bioreactor were investigated. The results showed that a maximum ginsenosides yield of up to 12.42 mg/g dry weight extract under appropriate conditions can be obtained after 5 weeks of culture [105]. Another study compared the properties of *P. ginseng* hairy roots between a flask and aerated column or stirred bioreactor, and the results showed that it was almost three times as high as the flask culture of both bioreactors [104]. Another essential factor for the bioreactors’ cell and root suspension cultures is the inoculum size, which can affect cell growth and secondary metabolite production [106,107,108]. Differences in the cell inoculum size can cause a significant difference in cell density during culture. Thus, it can lead to changes in culture conditions, such as the concentration of dissolved oxygen and gas metabolites, as well as the related enzyme activities, depending on the number of accumulated cells. These changes could affect cellular metabolism both directly and indirectly. There have been reports about the effect of inoculum size on cell growth and secondary metabolite accumulation [35,109,110,111], and the effect of cell density varies depending on the type of vessels and period of culture [112].

Faster biomass production increases the efficiency of producing secondary metabolites of economic interest. Therefore, using bioreactors to manufacture biomass in vitro is a novel strategy, frequently used to meet needs that are challenging to meet in the field because of pesticide use, climate change, and water restrictions. However, due to some limitations of plant tissue culture, it cannot be viewed as a replacement for actions to prevent or combat climate change. According to previous reports, the plantlets obtained from in vitro culture were initially small and had unfavorable traits. In vitro, plants must go through a transitional stage before independent growth because they cannot function autotrophically when cultured in vitro. The potential for creating plants with genetic aberrancy may increase. Plants are more vulnerable to contamination and water loss since they are grown in an atmosphere with high relative humidity [113].

Moreover, different cultivation conditions result in significant variations in quantitative and qualitative material characteristics originating from plants. Hence, the relationship between the propagation method and the quality of secondary metabolites should be made clear when using these technologies to obtain the most balanced cost–benefit ratio.

## 4. Perspectives on the Breeding of *P. ginseng* and Conclusions

Medicinal plants offer significant advantages for both people and the environment, and their functional and therapeutic values are higher than any other crop. However, the study and creation of therapeutic plants have been largely overlooked, and little is known about their genetic makeup, heterozygosity, growth patterns, and self-incompatibility [114]. This limitation further impedes the progress of medicinal plant breeding. Given the diversity of therapeutic plants and the environmental conditions in which they grow, breeding them is often an exceedingly intricate process. Therefore, collaborative efforts are necessary to address these challenges. The following sections will discuss current breeding strategies and future directions to overcome these challenges in *P. ginseng*.

### 4.1. Molecular Breeding

Molecular breeding is an essential content of molecular pharmacognosy. The process of breeding at the molecular level using molecular biology techniques is known as “molecular breeding”, which is a new development of conventional breeding. Conventional breeding emphasizes phenotypic selection, while molecular breeding focuses on genotype selection. Molecular breeding is inseparable from conventional breeding. It also takes excellent phenotypes as the breeding goal, establishes the connection between genotype and phenotype, and selects phenotypes by genotype. Medicinal plant breeding is a crucial method for improving the quality of medicinal materials. In the past 20 years, through systematic breeding, crossbreeding, polyploid breeding, and other conventional breeding methods, many medicinal materials have been cultivated, such as *P. ginseng*, *P. quinquefolium*, *Rehmannia glutinosa*, *Salvia miltiorrhiza*, *Platycodon grandiflorum*, *Magnolia officinalis*, etc. However, due to the variety of medicinal plants, long growth cycles, high heterozygosity, unique breeding objectives, and other reasons, the overall level of medicinal plant breeding and breeding efficiency is not high [115].

While some progress has been made in using molecular markers for studies of therapeutic plants, most research has concentrated on identifying species and genetic polymorphism. There have been relatively few reports on marker-assisted breeding for these plants [116]. For instance, the successful application of DNA-marker-assisted selection and systematic breeding is developing a new variety of *P. notoginseng* called “Miao Xiang Kang qi” [117]. In this case, specific single-nucleotide polymorphisms (SNPs) identified in resistant varieties associated with root rot resistance act as genetic markers to assist systematic breeding. The incidence of root rot and rust was reduced by 83.6% and 71.8%, respectively, compared with conventional varieties. There are few reports of DNA-marker-assisted methods for selecting new ginseng cultivars [118,119,120,121]. Molecular breeding is fast, efficient, and accurate. Therefore, it can be used as a new reference for breeding and to direct the breeding of new variations of *P. ginseng*. More DNA molecular markers should be exploited to direct the future development of *P. ginseng* breeding [122].

### 4.2. Transgenic Breeding

Transgenic breeding is a molecular method in which one or more foreign genes are transferred to a plant through genetic engineering so that the plant can effectively express the corresponding products. The basic principle of genetic modification (GM) is like that of conventional crossbreeding: crossbreeding involves the transfer of whole gene chains (chromosomes). In contrast, gene transfer involves the selection of the most practical small gene segments, so GM is more selective than crossbreeding. Since the establishment of the *Agrobacterium*-mediated method [123], gene gun-mediated method [124], and pollen tube channel method [125], significant achievements have been made in the transgenic breeding of crops and horticultural crops for disease resistance, insect resistance, and stress resistance.

However, the uncertain genetic background, high heterozygosity, and repetitive sequences of medicinal plants make transgenic breeding more challenging than in other crops. Nevertheless, there have been successful examples of genetically modified medicinal plants, such as *Artemisia annua*. Despite this, the research on transgenic breeding of medicinal plants remains limited [126]. The agrobacterium-mediated method has been studied predominantly for the synthesis of secondary metabolites. There is also limited research on the transgenic breeding of *P. ginseng*. An attempt has been made using the Agrobacterium-mediated method to produce herbicide-resistant transgenic *P. ginseng* plants via the introduction of the phosphinothricin acetyl transferase (PAT) gene that confers resistance to the herbicide Basta. The results showed that transgenic *P. ginseng* grown in soil exhibited high Basta resistance [127]. In another report, thermotolerant transgenic *P. ginseng* was produced by introducing the isoprene synthase gene through Agrobacterium-mediated transformation. The transgenic plant appeared healthy when exposed to a high temperature of 46 °C for 1 h. In contrast, the non-transformed ones were wilted from heat shock, which suggested that the exogenous isoprene synthase gene can be added as an alternative technique for producing thermotolerant ginseng [128]. There is no available research report on transgenic breeding of medicinal plants via the pollen tube method. More transgenic genes must be exploited to produce either high tolerance or secondary metabolites production *of P. ginseng* plants.

Genetic modification is a promising approach that enables us to understand regulatory mechanisms through genetic alterations of single or few genes. It is worth noting that plants obtained from transgenic performances are only sometimes uniquely responsive, especially when performing classical genetic improvement activities in the field. Some studies reported that the increased yield of transgenic plants was carried out in controlled greenhouse conditions, and the response to a particular transgene can be reversed in the field [129]. However, transgenic lines were unable to maintain the advantages observed under control settings in field testing [130].

### 4.3. Digital Breeding

Due to advancements in DNA sequencing technologies and bioinformatics, many crop genomes are now publicly available. While having a reference genome sequence (the size of *P. ginseng* species is 3.4 Gb according to GenBank accession number GCA_020205605.1 in NCBI) is valuable, it does not fully represent the genetic diversity within a particular species. Therefore, information on DNA polymorphisms is essential for crop breeding. Hence, techniques such as whole-genome resequencing [131], sequence capture, target enrichment, resequencing methods [132], partial genome sequencing strategies [133,134,135], and high-density genotyping arrays [135] are highly beneficial. Genetic diversity studies have recently been conducted on staple and “orphan” crops [135,136,137,138].

Bioinformatics is a rapidly growing research area due to the crucial importance of extracting knowledge from diverse data, known as data mining. Analyzing a large amount of SNP and phenotypic data requires sufficient computing infrastructure and bioinformatics and shell scripting expertise, which is not commonly available in laboratories. Furthermore, there is a rising need to combine various “omics” data, such as genomics and phenomics, with mathematical and statistical models.

Therefore, developing bioinformatics skills among plant researchers and breeders is critical to ensuring they can analyze and interpret their data [139]. However, finding individuals with bioinformatics and plant breeding skills is challenging. The best solution is to form an interdisciplinary team where researchers can share knowledge and skills to advance crop improvements. The first open-access platform to offer extensive genetic resources of *P. ginseng* was created by Murukarthick Jayakodi et al. The most up-to-date draft genome sequence is available in the current version of this database, along with 59,352 gene structural and functional annotations and digital expression of genes based on transcriptome data from various tissues, growth stages, and treatments [140]. In another report, Woojong Jang et al. revealed the plastome diversity and established a standard haplotype grouping system according to various genotypes of ginseng plastomes. Eighteen polymorphic sites were identified, among which 11 SNPs and 7 INDels are included, with the help of a comparative investigation of the plastomes of 44 cultivated and wild ginseng accessions from Northeast Asian nations. Based on the SNP variants, 10 KASP markers were created to identify various haplotypes and their cultivation histories in various genetic resources. The understanding of ginseng evolution is intensified by establishing a digital haplotyping approach based on plastome diversity, which also acts as a powerful molecular breeding tool [141].

In contemporary times, the skill sets of breeders are evolving rapidly, which are rich enough that it is time we start thinking about breeding with different tools than in the past. Because of technological advances in phenotypic and genotypic analysis, as well as in biotechnology and the digital revolution, breeding cycles will be shortened cost-effectively [142]. Therefore, we can consider these new tools for breeding *P. ginseng* in the future.

### 4.4. Conclusions

This review has summarized various in vitro cultivation methods via direct and indirect organogenesis technologies and the ginsenosides’ biosynthetic pathways. In addition, biotechnological approaches for ginsenosides accumulation, including adventitious root culture, cell suspension culture, hairy root culture, biotic and abiotic factors, and large scale-up culture for the high production of ginsenosides, have been explored. Despite significant advancements in ginseng in vitro culture, there is still more room for the research community to identify superior chemotypes of ginseng species for propagation. The perception of new techniques like transplanting seedlings and aeroponics culture methods are proposed as a significant requirement to grow high-quality ginseng rapidly. Finally, we have also introduced some breeding technologies, which may provide new insights as better options for prior cell lines of *P. ginseng* for better application and highly stable production of its secondary metabolites.

## Figures and Tables

**Table 1:** Ginseng species.

No.	Scientific Name	Common Name	Rank	Cultivation Area
1	*P. ginseng* C. A. Meyer	Korean ginseng, Ginseng	Species	China, Republic of Korea, Russia
2	*P. notoginseng* (Burkill) F. H. Chen	Chinese ginseng, Sanchi ginseng	Species	China
3	*P. quinquefolius*	American ginseng	Species	China, America, Canada
4	*P. japonicus* C.A. Meyer	Japanese ginseng	Species	China, Japan
5	*P. pseudoginseng* Wallich	Himalayan ginseng	Species	China, Nepal
6	*P. vietnamensis* Ha & Grushv	Vietnamese ginseng	Species	China, Vietnam
7	*P. stipuleanatus* H.T. Tsai & K.M. Feng	Not mentioned	Species	China, Vietnam
8	*P. trifolius* L.	Dwarf ginseng	Species	America, Canada, Germany
9	*P. zingiberensis* C.Y. Wu & K.M. Feng	Not mentioned	Species	China, Nepal, Bhutan, Myanmar
10	*P. wangianum* S.C. Sun	Not mentioned	Species	China
11	*P. assamocus* R.N. Banerjee	Not mentioned	Species	India
12	*P. variabilis* J. Wen	Not mentioned	Species	China, India
13	*P. omeiensis* J. Wen	Not mentioned	Species	Not mentioned
14	*P. sinensis* J. Wen	Not mentioned	Species	East Himalaya
15	*P. shangianus*	Not mentioned	Species	Not mentioned

**Table 2:** Callus induction and culture of *P. ginseng*.

Explants	Medium	PGRs		Other Factors	Ref.
		2,4-D	KT		
roots	MS	2 mg/L	0.5 mg/L		[39]
roots	MS	1 mg/L			[40]
roots	MS	2 mg/L		1 g/Lpeptone, 0.5 mg/L TDZ	[41]
roots	MS	1 mg/L	0.1 mg/L		[42]

## Data Availability

The data are contained within the article.
